# Unmasking the Neglected Cholera Outbreaks in Sub-Saharan Africa

**DOI:** 10.3389/ijph.2024.1607990

**Published:** 2025-01-07

**Authors:** Beenzu Siamalube, Emmanuel Ehinmitan

**Affiliations:** Department of Molecular Biology and Biotechnology, Pan African University Institute of Basic Sciences, Technology and Innovation, Nairobi, Kenya

**Keywords:** Sub-Saharan Africa, cholera endemic areas, economic instability, oral cholera vaccines, climatic factors

## Introduction

Cholera is a communicable infection that is predominantly transmitted by the bacterium *V. cholerae* after ingesting contaminated water or food. It causes excessive diarrhoea and vomiting that can result in severe dehydration. *V. cholerae* traces its natural habitat in waterlogged settings, therefore, to effectively manage the spread of cholera, it is critical to research the interactions of the disease’s causative agent with that of human comportment and the ecosystem [1]. Despite the momentous breakthroughs that have been documented in the medical field, cholera remains a public health threat in some countries of Sub-Saharan Africa (SSA) as well as the East Mediterranean Region (EMR), and it claims thousands of lives, whenever there is an outbreak. The health crises are persistent in regions that lack adequate water supply and experience poor sanitation and hygiene services (WASH) alongside substandard healthcare infrastructure [2]. However, to productively highlight the neglected cholera incidences that reemerge year after year, in SSA, it is important to carry out detailed investigations of their systemic limitations that propagate their reoccurrence [3]. Thereafter, coming up with sustainable solutions to widely address this ongoing crisis (Table 1).

**TABLE 1:** The persistent challenge of cholera in Africa: a complex interplay of climatic factors, Africa, 2024

Category	Key causes/patterns	Context in SSA
Structural Poverty and Inequalities	- Marginalized populations in urban slums and rural areas are underserved - Poverty forces reliance on contaminated water sources	- Limited political prioritization leaves communities without access to clean water or sanitation - Informal economies restrict resources for public health investments
Environmental and Climatic Vulnerabilities	- Seasonal floods contaminate water supplies - Droughts exacerbate water scarcity - Unplanned urbanization overwhelms waste management	- Rapid urban growth in informal settlements increases exposure to cholera - Climate extremes amplify risks of waterborne diseases in vulnerable regions
Conflict and Fragility	- Displacement due to armed conflicts leads to poor camp conditions - Fragile health systems fail to provide adequate cholera prevention	- Refugee and IDP camps often lack water, sanitation, and healthcare. - Reliance on emergency aid undermines sustainable solutions
Cultural and Behavioural Barriers	- Traditional practices hinder adoption of hygienic behaviours - Distrust in authorities limits acceptance of health interventions	- Hygiene education efforts are culturally insensitive or absent - Behavioural norms (e.g., open defecation) perpetuate unsafe practices
Gaps in Governance and Policy	- Inconsistent funding focuses on reactive measures instead of proactive investments - Poor collaboration across sectors (water, sanitation, health)	- Cholera response programs remain underfunded and poorly coordinated - Long-term investments in public health infrastructure are deprioritized
Cross-Border Transmission Patterns	- Porous borders and trade routes enable cholera spread across countries	- Frequent migration and regional trade increase risk of cross-border outbreaks
Neglected Investment in Preventative Measures	- Vaccination campaigns are limited and reactive - Health education efforts fail to address systemic barriers	- Oral cholera vaccines are underutilized - Public awareness campaigns are rare and lack cultural or contextual relevance

The social constraints such as gender inequalities contribute to the public health challenge that is posed by cholera due to water scarcity. Girls and women bear the burden of caregiving during outbreaks as they are usually in the forefront [4]. Healthcare expenditures tend to strain fragile economies and reduce productivity. The cost of cholera treatment in Africa varies significantly depending on factors such as WASH infrastructure, severity of outbreaks, and access to resources. In Somalia for instance, the overall average cost of cholera treatment for health facilities and households was estimated to be US$ 116.59 in 2023 [5]. This hefty cost considering the socioeconomic inequality, disproportionately affects the poor in society. Additionally, the young population below the age of five are highly susceptible, especially those that have little to no food to eat, the malnutrition renders disease vulnerability to the children, due to their compromised immune systems. Their bodies find it hard to defend themselves against foreign invaders [6]. Cholera epidemics repeatedly erupt during foreseeable periods in Africa, they frequently align with the rainy seasons and are intensified by environmental and infrastructural dynamics [7]. The infectious disease is remarkably prevalent in SSA, where countries such as Zambia, Nigeria and the Democratic Republic of Congo have succumbed to major episodes and recorded high case fatality rates [8]. According to the World Health Organization (WHO), the SSA reports the highest number of cholera cases, globally, the region records over 150,000 laboratory confirmed cases, and about 3,000 deaths annually [9].

In several countries of SSA, specifically in conflict-affected regions and rural areas, health centres are not fully equipped to execute timely diagnosis and reporting of cholera cases. As a result, most outbreaks remain masked to both national and international health authorities, until they escalate [10]. Political influences equally play a key role, as some governments may shy away from reporting cholera outbreaks for fear of ruining their international reputation or suppressing the tourism industry and discouraging investment. Additionally, the prolonged regional or internal conflicts contribute to the prevalence of cholera chapters in SSA. They disrupt the healthcare systems and cause limited access to quality medical services [11]. Furthermore, there are fragile systems of water supply and drainages, especially in rural settings [12]. Climate change including floods and droughts contaminate water sources [13]. Insufficient vaccination coverage, particularly in hard-to-reach districts is also a chief cause of cholera outbreaks in SSA [14]. The high population density in refugee camps together with the informal settlements in urban areas increase the transmission rate, turning small outbreaks into large-scale epidemics [15]. Similarly, in the EMR, cholera remains a pressing health concern, with Afghanistan, Pakistan, Haiti, and Yemen being among the most severely affected countries. The prevalence of cholera in these nations is driven by protracted conflicts, political instability, and widespread displacement, which disrupt healthcare and sanitation infrastructure [16].

In the period between 2010 and 2020, 34 African countries recounted cholera outbreaks [17]. The ten most affected countries being Ethiopia, the Democratic Republic of Congo (DRC), Somalia, Kenya, Mozambique, South Sudan, Nigeria, Tanzania, Zimbabwe and Uganda. Cholera endemic countries undergo a series of challenges when a crisis is neglected, such as high mortality rates [18], because cholera can kill within hours if left untreated [19] and economic instability that burden the commerce industry [20]. Similarly, high-risk communities face social exclusion and marginalization [21]. Also, cholera brings about long-term health consequences such as malnutrition [6], kidney damage and increased immunodeficiency. Notwithstanding, some of these countries have implemented strategic measures to spearhead the control and prevention of cholera transmission and in turn, break the cycle of neglect [22–26]. The innovative actions adopted by some countries include investing in improved WASH facilities [27], engaging in targeted vaccination programs, intensely in cholera hotspots [28] and empowering medical practitioners by training them how to diagnose and treat cholera patients. Public involvement has equally proven to be a pillar of cholera prevention, educating local communities in control and response efforts ensures disease awareness [29]. Alternatively, addressing underlying conflicts and providing emergency assistance can minimize the displacements of people and reduce the cross-border transmission [30].

To further mitigate cholera outbreaks in SSA and beyond, it is imperative for local stakeholders, governments, the international community, and non-governmental organizations to prioritize cholera control and prevention [31]. It would equally be of help to incorporate successful strategies that have been implemented in other regions. Ahmed *et al.* [32], reported that in March 2017, a team of specialists from ten Asian cholera at risk nations alongside various representatives from WHO came together in Vietnam to share progress in terms of prevention and control interventions on water, sanitation and hygiene (WASH), surveillance and oral cholera vaccine use [32]. This multi-sectoral collaboration can be implemented in SSA by integrating the Africa CDC’s “One Health” approach, to address broader factors contributing to cholera outbreaks. Besides, it is crucial to explore the WASH coverage indicators and their effect on the spread of cholera. Because the impact of inadequate WASH coverage on cholera in Africa contributes largely to the higher cholera incidence due to the ease of pathogen transmission [12]. Thus, improved WASH services, including access to safe drinking water, standard sanitation supplies, personal hygiene practices, and wastewater management, especially in cholera-prone areas and refugee camps would significantly reduce outbreaks by breaking the transmission cycle, enhancing prevention, and promoting community resilience [33].

Another method to upscale the alleviation of outbreaks in cholera-prone areas, would be the introduction of mass immunization movements using the Oral Cholera Vaccines (OCVs) [34]. A few African countries have initiated OCV campaigns in their respectful predisposed districts. Even though OCVs offer provisional protection, they are certainly an essential component in the fight against cholera. Likewise, local establishments and initiatives led by the community whose aim is to tackle triggers that bring about cholera outbreaks [35], could be supported by governments to unmask the overlooked threats on matters public health. In addition, the healthcare technologies need to be ameliorated to heighten cholera surveillance and medical response in the quest for combatting cholera outbreaks in SSA [36]. This involves developing standard early warning schemes that can detect outbreaks before they spiral out of control [37].

## Recommendations

**FIGURE 1:**
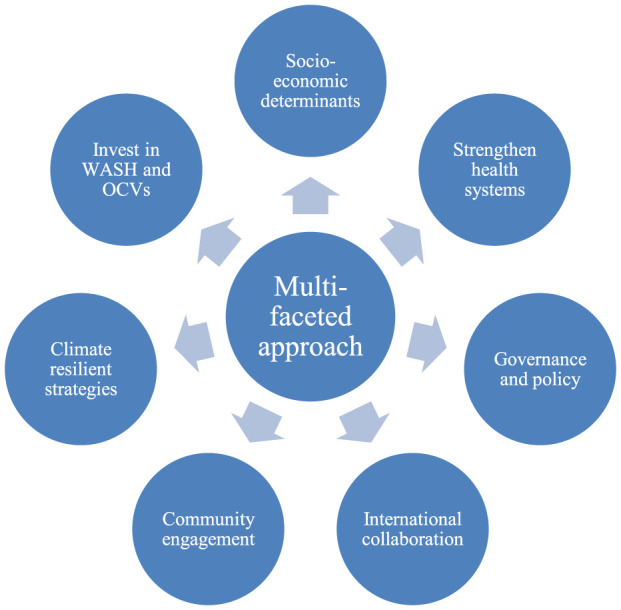
Cholera in Zambia: Explanatory factors and mid-term impact of the sustainable development goals, Zambia, 2024.

A multifaceted approach to address cholera as a public health problem in Africa should focus on tackling the root causes linked to socioeconomic inequalities. These disparities such as limited access to clean water, sanitation and healthcare are critical drivers of cholera outbreaks in vulnerable populations. Hence, addressing poverty and housing conditions are crucial to mitigating cholera risks, alongside involving policymakers and strengthening international collaboration. In the EMR, multisectoral collaboration is often led by international agencies due to weak local governance. Non-Governmental Organizations (NGOs) equally play a critical role in coordinating cholera responses across sectors in the EMR. Over-reliance on external actors limits the sustainability of such approaches. SSA’s focus on strengthening domestic multisectoral frameworks offers valuable insights for EMR, while EMR’s reliance on global partnerships highlights the importance of leveraging external support in resource-limited SSA countries (Figure 1). Ultimately, an effective approach must integrate health, education, and infrastructure efforts to address the systemic inequities that perpetuate cholera outbreaks in Africa.

### Conclusion

Cholera remains a persistent yet preventable public health crisis in Sub-Saharan Africa, exacerbated by socioeconomic inequalities, poor WASH infrastructure, and the impacts of climate change. Despite its devastating effects, cholera often receives inadequate attention and resources, leaving vulnerable communities disproportionately affected. Addressing this crisis requires a holistic approach that prioritizes investments in clean water and sanitation, strengthens healthcare systems, and implements climate-resilient strategies. Empowering local communities through education and proactive policies targeting poverty and inequality can ensure sustainable change. International collaboration is also crucial for funding and technical support. By unmasking the structural challenges underlying cholera outbreaks and committing to comprehensive solutions, the region can break the cycle of neglect and secure better health outcomes for all.
